# Identification of a novel pax8 gene sequence variant in four members of the same family: from congenital hypothyroidism with thyroid hypoplasia to mild subclinical hypothyroidism

**DOI:** 10.1186/1472-6823-14-69

**Published:** 2014-08-22

**Authors:** Monica Vincenzi, Marta Camilot, Eleonora Ferrarini, Francesca Teofoli, Giacomo Venturi, Rossella Gaudino, Paolo Cavarzere, Giuseppina De Marco, Patrizia Agretti, Antonio Dimida, Massimo Tonacchera, Attilio Boner, Franco Antoniazzi

**Affiliations:** 1 Department of Life and Reproduction Sciences, University of Verona, Piazzale Scuro 10, 37126 Verona, Italy; 2 Azienda Ospedaliera Universitaria Integrata di Verona, Verona, Italy; 3 Department of Endocrinology, Centro di Eccellenza AmbiSEN, University of Pisa, Pisa, Italy

**Keywords:** PAX8 gene, Thyroid, Congenital hypothyroidism, Variable phenotypic expressivity, R133W-PAX8

## Abstract

**Background:**

Congenital hypothyroidism is often secondary to thyroid dysgenesis, including thyroid agenesis, hypoplasia, ectopic thyroid tissue or cysts. Loss of function mutations in TSHR, PAX8, NKX2.1, NKX2.5 and FOXE1 genes are responsible for some forms of inherited congenital hypothyroidism, with or without hypoplastic thyroid. The aim of this study was to analyse the PAX8 gene sequence in several members of the same family in order to understand whether the variable phenotypic expression, ranging from congenital hypothyroidism with thyroid hypoplasia to mild subclinical hypothyroidism, could be associated to the genetic variant in the PAX8 gene, detected in the proband.

**Methods:**

We screened a hypothyroid child with thyroid hypoplasia for mutations in PAX8, TSHR, NKX2.1, NKX2.5 and FOXE1 genes. We studied the inheritance of the new variant R133W detected in the PAX8 gene in the proband’s family, and we looked for the same substitution in 115 Caucasian European subjects and in 26 hypothyroid children. Functional studies were performed to assess the *in vitro* effect of the newly identified PAX8 gene variant.

**Results:**

A new heterozygous nucleotide substitution was detected in the PAX8 DNA-binding motif (c.397C/T, R133W) in the proband, affected by congenital hypothyroidism with thyroid hypoplasia, in his older sister, displaying a subclinical hypothyroidism associated with thyroid hypoplasia and thyroid nodules, in his father, affected by hypothyroidism with thyroid hypoplasia and thyroid nodules, and his first cousin as well, who revealed only a subclinical hypothyroidism. Functional studies of R133W-PAX8 in the HEK293 cells showed activation of the TG promoter comparable to the wild-type PAX8.

**Conclusions:**

*In vitro* data do not prove that R133W-PAX8 is directly involved in the development of the thyroid phenotypes reported for family members carrying the substitution. However, it is reasonable to conceive that, in the cases of transcriptions factors, such as Pax8, which establish several interactions in different protein complexes, genetic variants could have an impact *in vivo*.

## Background

Congenital hypothyroidism (CH) is a common disease with a worldwide incidence of 1 in 3,000-4,000 newborns [1]. In 85% of the cases, CH is secondary to thyroid dysgenesis, including thyroid agenesis, hypoplasia, ectopic thyroid tissue or cysts [2].

Loss of function mutations of the thyrotropin receptor (TSHR) are responsible for some forms of recessively inherited congenital hypothyroidism, either with normal or hypoplastic glands [3]. Other cases of thyroid dysgenesis may result from mutations in some of the transcription factors genes involved in thyroid development, such as PAX8, NKX2.1 (also known as TTF1), FOXE1 and NKX2.5 [2,4-6].

Pax8 is a member of the large mammalian Pax protein family, a group of important developmental regulators defined by the presence of a highly conserved DNA-binding motif of 128 amino acids, the so-called “paired-box domain”. This element is well conserved during evolution and consists of two distinct structurally independent subdomains, each containing a helix-turn-helix motif, joined by a linker region [7]. The N- and C- terminal subdomains are called PAI and RED, respectively [8].

During mouse embryogenesis, PAX8 gene is expressed in the developing thyroid, in the kidney and in several areas of central nervous system [9-11]. In addition to its role in thyroid development, Pax8 is an important regulator of thyroid differentiation through the activation of specific genes expression, namely thyroid peroxidase (TPO) and thyroglobulin (TG) [12,13].

In humans, PAX8 gene maps to chromosome 2q12-q14 and consists of at least ten exons [14]. So far, several PAX8 mutations and a rare sequence variant have been reported [2,15-27]. They have been mostly detected in patients with normally located but hypoplastic thyroid gland, often associated with renal anomalies, and in a few patients with complete athyreosis [2,16-19,21]. The aim of this report is to analyse PAX8 gene in members of the same family with variable phenotypic expressivity: from congenital hypothyroidism with thyroid hypoplasia, to mild subclinical hypothyroidism, in order to establish whether a correlation between variants in the PAX8 gene and different phenotypes is present.

## Methods

### Subjects

**Figure 1:**
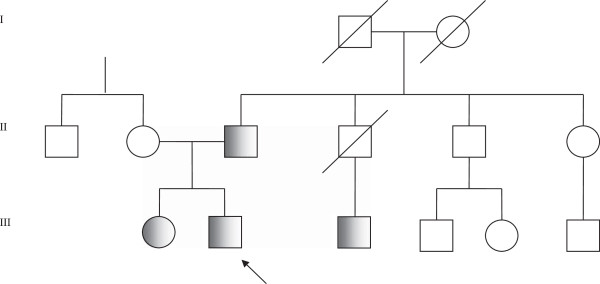
**Pedigree of the family discussed.** The arrow indicates the proband. The R133W carriers are evidenced in grey. The subjects II-2, II-5, II-6, III-4, and III-5 were subject to DNA analysis and they did not carry the R133W variant.

In this study we analysed 9 members of a family with history of hypothyroidism, 26 patients with congenital hypothyroidism and 115 healthy subjects.Patient III-2 (Figure 1), the proband, a male subject, was born in 1983 after an uneventful pregnancy. The patient was diagnosed as hypothyroid in the reference centre for newborn screening programs of North-Eastern Italy. TSH and total T4 values, assayed in dried blood spot collected at 3–5 days of life by radioimmunoassay, were 281 mIU/L (controls <10 mIU/L) and 18 nmol/L (controls >60 nmol/L), respectively.

Serum investigations confirmed the diagnosis of congenital hypothyroidism (TSH 93 mIU/L and free T4 17 pmol/L, normal values 0.4-4.0 mIU/L and 10.3-24.5 pmol/L respectively), and substitution therapy with L-thyroxine 37 μg daily was started at three weeks of life. The dose was adjusted to 50 μg/d at four months of life, and was stabilized at 75 μg/d when the infant was ten months old. He is now assuming 125 μg daily. The ultrasound examination demonstrated a hypoplastic but normally located thyroid gland. Abdomen ultrasound investigation showed no abnormalities. His growth, renal function (at blood tests), neuropsychological development and IQ (measured several times and with different test depending on the age) were normal. During one of the follow-up examinations and after obtaining written informed consent, peripheral blood samples in EDTA were collected from the patient and his parents in order to screen for mutation in the PAX8, TSHR, FOXE1, NKX2.1, NKX2.5 genes.

Upon detection of the same PAX8 sequence variant detected in the index case, subject II-3, father of the proband, was submitted to endocrinological examination; at that time he was 60 years old. Serum determinations evidenced TSH concentration of 20.5 mIU/L (normal values 0.4-4.0 mIU/L) and free T4 level of 9.4 pmol/L (n.v. 10.3-24.5 pmol/L). Thyroid autoantibodies were negative and serum thyroglobulin was in the normal range. Substitutive therapy with L-thyroxine 50 μg daily was promptly started. After four months of therapy, plasma TSH decreased to 7.39 mIU/L (n.v. 0.35-5.50 mIU/L) and free T4 raised to 14.7 pmol/L (n.v. 11.5-22.7 pmol/L). Substitutive therapy was changed to 50–75 μg every other day, and both serum TSH and free T4 returned within the corresponding ranges of the reference population. Despite the lack of substitutive therapy until the age of 60, the physical and intellectual development was normal. He did not present neurological alterations. Renal function was in the normal range for age. Ultrasound examination revealed a normally located thyroid gland with hypoplasia of the left lobe (measuring 10 × 5 mm), and the presence of three nodules with calcifications (measuring 15 × 16 mm) in the right one. A fine needle aspiration biopsy was performed on the right lobe. A moderate colloid amount and some aggregates of thyroid cells with hypertrophic nucleus were detected. Malignant cells search was negative. Thyroid scintigraphy revealed poor uptake. The presence of the isthmus was not reported on ultrasound examination, and it was not on uptake scan.

Seven members of the same family (subjects II-2, II-5, II-6, III-1, III-3, III-4, III-5) were asked to send us capillary blood samples absorbed on paper (Schleicher & Schuell 903 paper), in order to search for the same PAX8 variant. Subject III-1, the proband’s sister, aged 30 years, revealed to be a carrier of the PAX8 sequence variant. Her serum TSH was 6.85 mIU/L (n.v. 0.25-3.50 mIU/L) and serum free T4 was 16.9 pmol/L (n.v. 10.3-24.4 pmol/L). Thyroid autoantibodies were negative. Serum thyroglobulin was 244.00 ng/mL (upper limit 60.00 ng/mL). The ultrasound investigation evidenced a thyroid gland located in the normal position, with isthmus agenesis, hypoplastic left lobe and right one with three nodules. A fine needle aspiration biopsy was performed on the right lobe. No abnormalities were reported at the cytological analysis. Abdomen echography showed no anomalies. No neurological or renal dysfunctions were evidenced. Her condition was periodically monitored and L-thyroxine therapy was no indicated, since her hypothyroidism remained subclinical. As for the father (subject II-3), the daughter (III-1) came to our attention undetected from birth, because at the time of their birth newborn screening for congenital hypothyroidism still had to be instituted.

Patient III-3, cousin of the proband, showed to be a carrier of the PAX8 sequence variant too. His serum TSH value is mildly elevated (4.66 mIU/L, n.v. 0.27-4.20 mIU/L) with a fT4 in the normal range. Thyroid autoantibodies were negative and serum thyroglobulin was 95.70 ng/mL (upper limit 60.00 ng/mL). His thyroid function was periodically checked, evidencing a persistent subclinical hypothyroidism. At the moment he takes no therapy. He was submitted to thyroid ultrasound that evidenced a normally located thyroid gland with both the lobes slightly reduced in size. No other clinical abnormalities were found. Notably, he reported that his father (patient II-4, proband’s uncle) was treated with L-thyroxine for one year before cardiac surgery. He died of heart failure.In all the other relatives reported in Figure 1 thyroid function was evaluated and TSH, fT4 and fT3 values were in the normal range. No relevant clinical alterations were described for them. Given the family history, we recommended to these unaffected family members a periodic follow-up. At the moment, we have no information of alterations in their thyroid function.

In order to exclude the occurrence of the new PAX8 genetic variant as a common polymorphism, 115 healthy Caucasian European subjects have been screened for the same substitution. They signed written informed consent for the genetic analysis.

In addition, 26 patients with congenital hypothyroidism followed at Pediatric Endocrinology Division of Verona Hospital during 2011, were screened for the same substitution, after their parents’ written consents were obtained. All of them were older than fourteen and were in L-thyroxine treatment from birth. Thirty-one per cent of them did not present echographic alterations, 25% showed thyroid hypoplasia, 31% ectopia and the remaining 13% agenesis of the gland.

The study was conducted in compliance with the terms of the Helsinki II Declaration and written informed consent for the enrolment and for the publication of individual clinical details was obtained from patients or, whenever participants were children, from their parents or guardians.

In our country, namely Italy, this type of clinical study does not require Institutional Review Board/Institutional Ethics Committee approval to publish the results.

### Genetic analysis

Genomic DNA was extracted from peripheral venous blood on EDTA by means of the Gentra Puregene Blood kit (QIAGEN S.p.A, Milan, Italy), following the manufacturer’s instructions.

Genomic DNA was extracted from the blood spot paper cards by means of the QIAamp® DNA Micro kit (QIAGEN S.p.A., Milan, Italy), according to the manufacturer’s instructions.

We amplified by PCR all the exons of PAX8, TSHR, FOXE1, NKX2.1, NKX2.5 genes by means of intronic primers. Fragments were first analyzed by Denaturing High Performance Liquid Chromatography on a WAVE DNA Fragment Analysis System (Transgenomic, Omaha, NE), and sequenced on an automated CEQ 8800 Genetic Analysis System (Beckman Coulter GmbH, Germany) whenever a sequence variation was suspected. PCR conditions, partial denaturing temperature (t_pd_) for DHPLC analysis and sequencing conditions for TSHR and PAX8 genes have been previously described [28]. For FOXE1, NKX2.1 and NKX2.5 genes, sequencing conditions are available upon request.

### Polyphen prediction

PolyPhen (=*Poly*morphism *Phen*otyping) is an automatic tool for prediction of possible impact of an amino acid substitution on the structure and function of a human protein. This prediction is based on straightforward empirical rules which are applied to the sequence, phylogenetic and structural information characterizing the substitution [29]. PolyPhen-2 is a new development of the popular PolyPhen tool and is available as freeware at http://genetics.bwh.harvard.edu/pph2/ web site.

### Construction of the expression vector and functional analysis

The wild-type PAX8 protein (WT-PAX8) was expressed in the vector pcDNA3 as already described [20].

Human thyroglobulin promoter was cloned in the pGL3 luciferase report vector (TG prom-pGL3), designed to provide enhanced reporter gene expression. TTF1-pcDNA3 and pCMV-HA-p300 expression vectors have been previously described [20]. pRL-TK, expressing Renilla luciferase activity, (Promega Corporation, Madison, WI) was used as internal control vector.

Mutant harbouring the single nucleotide missense substitution (R133W) was generated by site-directed mutagenesis using the GeneTailor site-directed mutagenesis system (Invitrogen Life Technologies, Carlsbad, CA). The accuracy of the recombinant construct was verified by direct sequencing.

HEK293 cells were grown in DMEM supplemented with 2 mM L-glutamine, 25 mM D-glucose, 50 U/mL Penicillin, 50 μg/mL Streptomycin and 10% FBS (Invitrogen Life Technologies, Carlsbad, CA) and plated in 12-well plates (2×10 [5] cells for well) for 24 h before transfection. Transfection was carried out with FuGENE 6 reagent, following the manufacturer’s instructions (Roche Diagnostic Corporation, Indianapolis, IN, USA), with a total amount of plasmid DNA of 1170 ng per well.

Cells were harvested 48 h later and analysed sequentially for firefly and *Renilla* luciferase activities by Dual-Luciferse Reporter Assay System (Promega Corporation, Madison,WI). The luminescence production in cell extracts was assayed using the Lumino luminometer (Stratec Electronic; GMBH, Birkenfeld, Germany). Light intensity was quantified using a pre-produced standard curve and was reported in relative light units (RLUs). The assay was performed in triplicate.

### Statistical analysis

Values of transcriptional activity of R133W mutant were compared with respect to the values obtained for wild type Pax8 using the Student two tails t-test. To test the significance, the risk level (p) was set at 0.05.

## Results

### Genetic analysis

**Figure 2:**
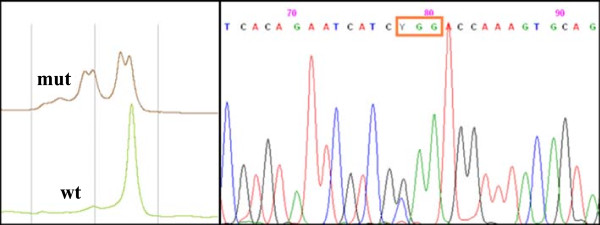
***In the left side: *DHPLC profiles of wild type (green) and mutated (brown) PAX8 exon 5.** The partial denaturing temperature was set at 61.3°C and the chromatographic parameters were obtained by means of the WAVEMaker software (Transgenomic, Omaha, NE), based on the amplicon sequence [23]. *In the right side:* Sequencing electropherogram of exon 5. A heterozygous C → T transition is shown where a Y is reported, corresponding to nucleotide 397 of the PAX8 coding sequence (NCBI Reference Sequence NM_003466.3). The mutation replaces a conserved arginine at position 133 with a tryptophan residue (R133W).

DHPLC analysis and direct sequencing of TSHR, FOXE1, NKX2.1 and NKX2.5 genes in genomic DNA of the patient III-2 revealed no variation compared to the NCBI Reference Sequences NM_000369.2, NC_000009.11, NC_000014.8 and NG_013340.1, respectively.During DHPLC analysis, a nucleotide substitution was suspected in the PAX8 fifth exon and its direct sequencing revealed the presence of a heterozygous transition of cytosine to thymine (C → T) at position 397 of the coding sequence (NCBI Reference Sequence NM_003466.3), leading to a change of a conserved arginine at codon 133 to tryptophan (c.397C/T, R133W) (Figure 2). The mutation was detected in the paired domain, the highly conserved PAX8 DNA-binding motif, at the end of the third alpha helix of the RED subdomain. This sequence variant was first detected in the propositus, affected by congenital hypothyroidism with a hypoplastic thyroid. The same substitution was present at the heterozygous state in his father, in his sister and in his first cousin, who evidenced mild hypothyroidism the first, and subclinical hypothyroidism the other two. In both the father and sister on the index case, the morphology of the thyroid showed hypoplasia and the presence of thyroid nodules.

We accessed the NCBI Single Nucleotide Polymorphism database and R133W was not found as a common variant. Moreover, the R133W variant was not present in a screen of 115 Caucasian European subjects (230 control chromosomes), reducing the likelihood that this substitution could represent a neutral common variant.

In addition, the PAX8 fifth exons of 26 hypothyroid children were analysed and none carried the R133W variant.

### PolyPhen prediction

The Polyphen Tool forecasted for the R133W variant a hydrophobicity change at buried site. Accordingly, R133W was predicted to be a pathogenic variant.

### Activation of TGprom-pGL3 by WT-PAX8 and R133W-PAX8

**Figure 3:**
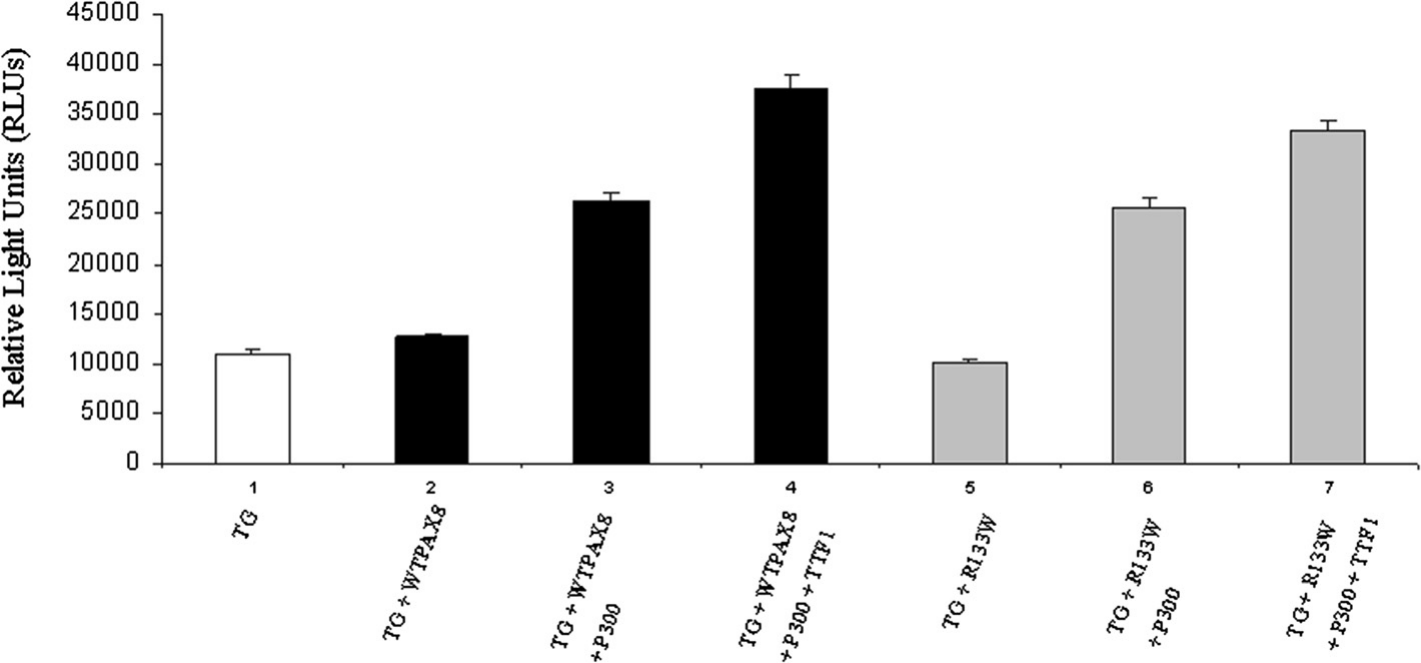
**The intensity of luminescence production in HEK293 cells is reported in relative light units (RLUs).** Firefly luciferase activities were normalized to the Renilla luciferase activity derived from cotransfected pRL-TK internal control vector (see text).

pCMV-HA-p300 was cotransfected with expression vector carrying WT-PAX8 or R133W-PAX8, together with an internal control (pRL-TK *Renilla*) and the TG prom-pGL3 (Firefly). The firefly to Renilla luminescence activity ratios were calculated and compared between groups. The transfection of WT-PAX8 together with 500 ng pCMV-HA-p300 revealed a significant increase in the TG promoter activity compared to WT-PAX8 alone (p < 0.01, Figure 3, columns 2–3), and this in accordance to literature [20,30]. R133W-PAX8, when transfected with pCMV-HA-p300, showed a significant increased TG promoter activity, to the same extent of WT-PAX8 (p < 0.01, Figure 3, columns 5–6 compared to 2–3). Cotransfection of TTF1-pcDNA3 resulted in a synergistic effect of p300 and the R133W-PAX8 or WT-PAX8 on the TG promoter (Figure 3 columns 4 *vs* 7 showed no statistical difference, 0.05 < p < 0.1).

## Discussion

The Pax8 transcription factor is required for mammalian morphogenesis of the thyroid gland, and it is essential for the thyrocite-specific promoter activation of the TPO and TG genes [9-11]. In humans, PAX8 mutation carriers have been reported to be hypothyroids with thyroid hypoplasia [2,15-27]. Most of the known mutations are monoallelic and localized in the paired box domain. They evidence a functional DNA-binding impairment, suggesting that hypothyroidism could be secondary to PAX8 haploinsufficiency.

In the present report we describe different members of the same family carrying the same PAX8 variant at the heterozygous state: the R133W substitution, replacing the last conserved arginine of the DNA-binding paired-box domain with a tryptophan residue, and characterized by a prediction of likely being a damaging substitution. The family members carrying the substitution evidence a remarkable phenotypic variability, from congenital hypothyroidism associated with thyroid hypoplasia, to mild subclinical hypothyroidism with thyroid hypoplasia and nodules.

In order to verify the actual role of the R133W variant in the development of the thyroid phenotype, we carried out *in vitro* functional studies in the TG promoter, known to be a genetic target of the Pax8 transcription factor. On the TG promoter, the Pax8 protein interacts with several other transcription factors in the formation of complexes targeting several regulatory regions [20]. The general transcriptional coactivator p300 has been shown to be essential in mediating Pax8 activation on both the TG and the rat thyroperoxidase (TPO) promoters. It plays a crucial role in the functional synergism between Pax8 and NKX2.1 in thyroid specific gene expression [20,30]. In order to assess whether R133W mutation was able to efficiently recruit p300 and assemble the transcriptional coactivation complex [20], we employed HEK293 cells. These cells, indeed, are deficient in endogenous p300 because of expression of the adenovirus E1A protein [30,31], which sequestrates p300 into the cytosol.

Functional studies of R133W-PAX8 in the HEK293 cells show comparable activation of the TG promoter of both the mutated and the wild-type Pax8. Furthermore, R133W-Pax8, similarly to the wild-type, is able to recruit p300 to synergistically transactivate the TG promoter.

Evaluation of 282 alleles belonging to either healthy or hypothyroid subjects revealed that R133W-PAX8 does not seem to be a common polymorphism and therefore it is probably a rare variant.

It is well known that persistent stimulation by increased plasma TSH levels leads to thyroid proliferation and often nodule formation [32,33]. This clinical observation could account for the similar phenotype of subjects II-3 and III-1, evidencing thyroid nodules, and for the substantial difference in the propositus, patient III-2, who was treated from birth with substitutive therapy, and displayed no thyroid nodules, at least so far.

It is well known in literature that often PAX8 gene mutations display variable expressivity, and even the same mutation gives rise to different clinical and biochemical phenotypes among members of the same family [16,18,19]. Our *in vitro* data does not prove that R133W-PAX8 is directly involved in the development of the phenotypes reported for the family members carriers of the aminoacidic substitution and, in this view, it could be a non influential polymorphism. However, since the crystallographic data of the last 4 residues of the PAX8 paired domain, corresponding to the region where the substitution R133W is located, still have to be completely clarified [7,34,35], we cannot exclude that interactions with other transcription factors could be responsible for the variable phenotypic expressivity evidenced in the reported family.

## Conclusions

Although *in vitro* data do not prove that R133W-PAX8 is directly involved in the development of the different thyroid phenotypes reported for the heterozygous carriers of the same family, it is reasonable to conceive that the substitution described could have an impact *in vivo*, the condition where all the interactions with different protein complexes actually take place. For transcription factors such as Pax8, indeed, genetic, epigenetic and environmental factors are likely involved, and only a thorough understanding of all the possible actors entangled can deeply clarify the actual contribution of variant such as the R133W-PAX8.

## Competing interests

The authors declare that they have no competing interests.

## Authors’ contributions

All the authors had full access to all of the data in the study and take responsibility for the integrity of the data and the accuracy of the data analysis. Moreover, all authors read and approved the final manuscript. MV conceived of the study, carried out the genetic analysis, contributed to the preparation and critical review of the manuscript. MC carried out the genetic and molecular analysis, contributed to the critical review of the manuscript. EF carried out the functional studies of the PAX8 gene variant. FT carried out the genetic and molecular analysis, contributed to the critical review of the manuscript. GV carried out the DNA extraction from control subjects. RG contributed to the recruitment of participants. PC helped to draft the manuscript. GDM carried out the molecular analysis. PA carried out the molecular analysis. AD carried out the molecular analysis. MT participated in the design of the study and in the coordination of genetic analysis and contributed to the critical review of the manuscript. AB participated in the design of the study and contributed to the critical review of the manuscript. FA conceived the study and participated in its coordination.

## Pre-publication history

The pre-publication history for this paper can be accessed here:

http://www.biomedcentral.com/1472-6823/14/69/prepub
